# Small airway dysfunction on impulse oscillometry and pathological signs on lung ultrasound are frequent in post-COVID-19 patients with persistent respiratory symptoms

**DOI:** 10.1371/journal.pone.0260679

**Published:** 2021-11-29

**Authors:** Agnaldo José Lopes, Patrícia Frascari Litrento, Bruna Cuoco Provenzano, Alícia Sales Carneiro, Laura Braga Monnerat, Mariana Soares da Cal, Angelo Thomaz Abalada Ghetti, Thiago Thomaz Mafort

**Affiliations:** 1 Department of Pulmonology, Piquet Carneiro Policlinic, State University of Rio de Janeiro (UERJ), Rio de Janeiro, Brazil; 2 Medical Sciences Post-Graduation Programme, School of Medical Sciences, State University of Rio de Janeiro (UERJ), Rio de Janeiro, Brazil; 3 Rehabilitation Sciences Post-Graduation Programme, Augusto Motta University Center (UNISUAM), Rio de Janeiro/RJ, Brazil; Ospedale S. Corona, ITALY

## Abstract

**Background:**

Thousands of people worldwide are suffering the consequences of coronavirus disease-2019 (COVID-19), and impulse oscillometry (IOS) and lung ultrasound (LUS) might be important tools for the follow-up of this population. Our objective was to prospectively evaluate abnormalities detected using these two methods in a cohort of COVID-19 survivors with respiratory symptoms.

**Methods:**

In this follow-up study, 59 patients underwent clinical evaluations, spirometry, IOS and LUS in the 2nd (M1) and 5th (M2) months after diagnostic confirmation of COVID-19 by real-time reverse transcriptase–polymerase chain reaction. Aeration scores were obtained from the LUS exams based on the following findings: B-lines >2, coalescent B-lines, and subpleural consolidations.

**Results:**

Fifty-nine (100%) participants had cough and/or dyspnea at M1, which decreased to 38 (64.4%) at M2 (p = 0.0001). Spirometry was abnormal in 26 (44.1%) and 20 (33.9%) participants at M1 and M2, respectively, although without statistical significance (p = 0.10). Normal examination, restrictive patterns, and obstructive patterns were observed in 33 (55.9%), 18 (30.5%), and 8 (13.6%) participants, respectively, at M1 and in 39 (66.1%), 13 (22%), and 7 (11.9%) participants at M2 (p = 0.14). Regarding IOS, considering changes in resistive and reactive parameters, abnormal exams were detected in 52 (88.1%) and 42 (71.2%) participants at M1 and M2, respectively (p = 0.002). Heterogeneity of resistance between 4 and 20 Hz >20% was observed in 38 (64.4%) and 33 (55.9%) participants at M1 and M2, respectively (p = 0.30). Abnormal LUS was observed in 46 (78%) and 36 (61%) participants at M1 and M2, respectively (p = 0.002), with a reduction in aeration scores between M1 and M2 [5 (2–8) vs. 3 (0–6) points, p<0.0001].

**Conclusions:**

IOS and LUS abnormalities are frequent in the first 5 months post-COVID-19 infection; however, when prospectively evaluated, significant improvement is evident in the parameters measured by these two methods.

## Introduction

The lungs are the organs most affected by coronavirus disease 2019 (COVID-19) [1]. The entry of severe acute respiratory syndrome coronavirus 2 (SARS-CoV-2) into human cells is facilitated by angiotensin-converting enzyme 2 (ACE2) receptors expressed by type 2 pneumocytes, leading to the release of antiviral cytokines in alveolar septa and the pulmonary interstitium [2]. Thus, extensive injury to epithelial and endothelial cells may occur, with secondary fibroproliferation, indicating a potential for chronic vascular and alveolar remodeling [3]. The aggressive action of the virus and its consequences on the lungs and blood vessels therefore raise concerns about the damage caused and the need to evaluate possible respiratory sequelae in survivors of COVID-19, as corroborated by data indicating that the disease burden after acute SARS-CoV-2 infection is very high [4].

Computed tomography (CT) of the chest has been widely used to determine the extent of the damage caused by SARS-CoV-2, although it is costly, has low availability, and exposes the patient to ionizing radiation, which limits its use in some populations [5]. In this scenario, lung ultrasound (LUS) in the evaluation of patients with COVID-19 has been increasingly used and should be encouraged because it is a practical, low-cost, and radiation-free method, in addition to requiring equipment that is easy to clean [5–7]. With the increase in the number of studies, LUS has been shown to be a useful tool to monitor lung disease progression in patients with COVID-19, especially considering that SARS-CoV-2 preferentially affects peripheral areas of the lungs where visualization of LUS signs is quite satisfactory [8, 9]. In addition, compared to CT, LUS appears to have an excellent discrimination capacity for the identification of pulmonary sequelae in post-COVID-19 follow-ups [7].

Different types of respiratory functional assessments can be performed objectively, with spirometry and diffusing capacity for carbon monoxide (DLco) being the most commonly used pulmonary function tests (PFTs) [1]. However, the use of these techniques during the pandemic raises concerns because of the risk of infection transmission through the generation of aerosols and coughing during such tests [10]. Given this concern, a simple method that does not require a forced expiratory maneuver to evaluate respiratory mechanics is impulse oscillometry (IOS), which also has the advantage of having high sensitivity to evaluate peripheral airway disease (PAD) [11]. IOS measures respiratory system impedance (Zrs), which consists of respiratory system resistance (Rrs, in-phase response) and respiratory system reactance (out-of-phase response) [12].

Because detecting pulmonary changes is essential in the diagnosis and follow-up of patients with respiratory sequelae caused by COVID-19, the use of measures of structural and functional abnormalities becomes essential in the patient population with pulmonary impairment caused by SARS-CoV-2. Given the heterogeneity of the clinical presentation of COVID-19, simple and sensitive tools are essential to monitor the impact of the disease on the respiratory system, especially considering the large number of survivors of COVID-19 requiring follow-ups [13]. Considering the various advantages that IOS and LUS offer at this time of the pandemic, the objective of this study was to prospectively evaluate abnormalities detected by these two methods in the 2nd and 5th months after a confirmed diagnosis of SARS-CoV-2 infection in survivors with respiratory symptoms.

## Methods

### Study design and participants

This is a prospective follow-up study of 59 consecutive patients (among 72 eligible patients) treated at Piquet Carneiro Policlinic, State University of Rio de Janeiro, Rio de Janeiro, Brazil, between October 10, 2020, and June 25, 2021. Patients aged ≥18 years with a previous diagnosis of COVID-19 confirmed by real-time reverse transcriptase–polymerase chain reaction (RT–PCR) 2 months prior who still presented with respiratory symptoms (cough and/or dyspnea based on patient self-reporting) were included. All patients had a previous diagnosis of COVID-19 pneumonia confirmed by CT scans at the time of acute SARS-CoV-2 infection. The following exclusion criteria were adopted: positive RT-PCR test at the time of inclusion in the study; smoking burden ≥10 pack-years; a history of lung resection; and an inability to perform acceptable maneuvers in PFTs (spirometry and/or IOS).

The study was approved by the Brazilian National Research Ethics Commission under number CAAE-30135320.0.0000.5259 and was conducted in accordance with the principles of the Declaration of Helsinki. All participants signed an informed consent form.

At 2 months (M1) and 5 months (M2) after the diagnosis of acute SARS-CoV-2 infection, the patients underwent clinical examinations, IOS, spirometry, and LUS.

### Clinical and anthropometric data

The following data were obtained by interview at M1: sex, age, weight, height, and body mass index (BMI). The history of comorbidities was based on patient reporting. Drug use, intensive care unit management, and CT results during the acute phase of SARS-CoV-2 infection were obtained through a retrospective review of medical records.

### Measurements

IOS was performed using an impulse oscillometer (Quark i2m, Cosmed, Rome, Italy). During the IOS evaluation, the participants were instructed to remain seated while maintaining the head in a neutral position and supporting the cheeks with the hands, with the nostrils closed by a clip, and to then breathe normally for 40 seconds [14]. The minimum acceptable coherence values were ≥0.9 Hz [15]. At least three trials were performed in accordance with the standard recommended by the European Respiratory Society [16], and the averages of three technically acceptable Zrs measurements from each subject were retained for further analysis [17]. The following resistive and reactive parameters were evaluated: Rsr at 4 Hz (R4), 6 Hz (R6), 10 Hz (R10), and 20 Hz (R20); the mean resistance between 4–20 Hz (Rm); the heterogeneity of resistance between R4 and R20 (R4-R20); resonance frequency (Fres); and the area under the reactance curve (AX). The R4, R6, R10, and R20 values were considered abnormal when ≥150% of the predicted value [18, 19]. AX was calculated as the integrated low-frequency reactance between 4 Hz and the Fres [20]. Fres and AX were considered abnormal when >12 Hz and >3.60 cm H_2_O/L/s, respectively [19]. R4-R20 was measured by calculating the difference between resistance at 4 Hz (total airway resistance including central and distal airway resistance values) and that at 20 Hz (large airway resistance), thus representing the resistance of small airways. For interpretation purposes, we also express R4-R20 as a relative percentage as follows [20]:

$$R4-R20=\frac{\left(R4-R20\right)}{R4}*100$$

An R4-R20 value ≥20% was operationally defined as abnormal and used for the diagnosis of PAD [20]. Because our IOS device allows measurement of the resistance at 4 Hz—which presumably better reflects the total airway resistance without requiring the suspension of spontaneous breathing [16]—we used the R4-R20 value, in line with most recently published studies [20, 21].

Immediately after a rest period of approximately 5 minutes following IOS, the participants underwent spirometry using a computerized system (nSpire Health, Inc., Longmont, CO, USA) in accordance with preestablished recommendations [22]. The Brazilian reference values were used when interpreting the spirometry results [23]. Obstructive disorder was defined as a forced expiratory volume in one second/forced vital capacity (FVC) <70%, while restrictive disorder was inferred as an FVC <80% of the predicted value in the absence of reduced expiratory flow [24].

Finally, the participants underwent LUS with an Aplio XG ultrasound machine (Toshiba Medical Systems, Tokyo, Japan) coupled to a 7.5–10-MHz multifrequency linear transducer or a 3.5–5-MHz convex transducer in B mode. All LUS evaluations were performed by the authors who, although not radiologists, are all employees of the Polyclinic and have at least 9 years of experience with LUS at the participants’ screening site. All LUS evaluations were performed by two examiners, and disagreements were resolved through collective discussion. With the participants in a sitting position, LUS signs were captured in six areas of each hemithorax as follows [25]: two anterior, two lateral, and two posterior. In the evaluation of the LUS pathological signs, we sought to identify B-lines >2, coalescent B-lines, and subpleural consolidations [8]. To obtain the aeration score, points were assigned to each of the six areas as follows: B-lines >2, 1 point; coalescent B-lines, 2 points; and consolidations, 3 points. The sum of all areas represented the aeration score [9].

### Statistical analysis

Nonparametric methods were applied because the variables did not show a Gaussian distribution based on the rejection of the normality hypothesis by the Shapiro-Wilk test together with graphical analysis of the histograms. Inferential analysis was performed by evaluating the variations in clinical data, PFT findings, and LUS findings between M1 and M2 (the absolute deltas for these measurements between M1 and M2 were also calculated). For this purpose, the Wilcoxon signed-rank test was used for numerical data, and the exact McNemar test was used for categorical data. Data analysis was performed using SAS 6.11 (SAS Institute, Inc., Cary, NC, USA). The results are expressed as the medians and interquartile ranges or frequencies (percentages), and statistical significance was considered when p < 0.05.

## Results

**Fig 1:**
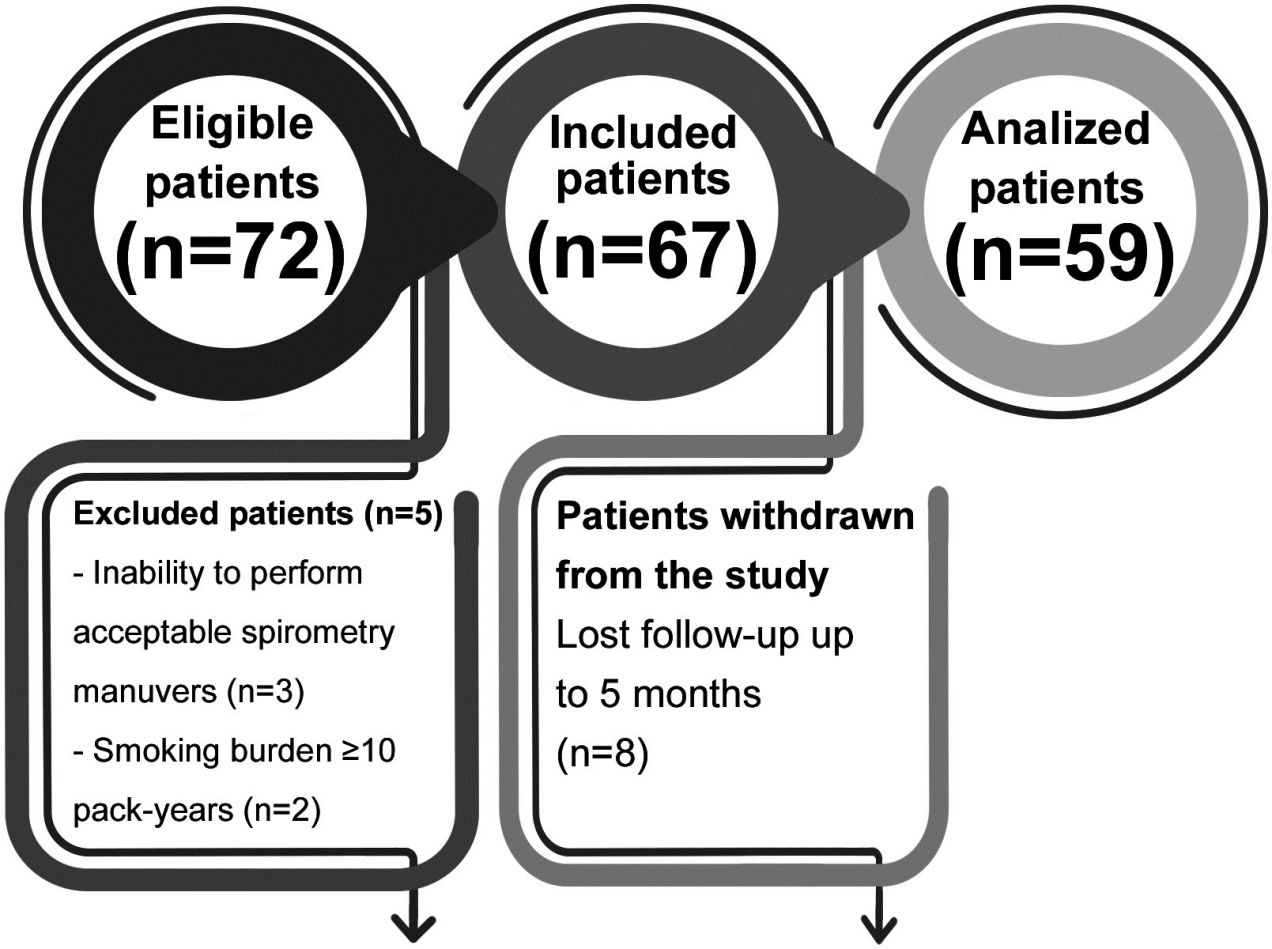
Flowchart showing exclusions and withdrawals during the study.

Of the 72 participants initially recruited for the study, 5 were excluded. Additionally, 8 patients were withdrawn from the study because they were lost to follow-up for 5 months. Thus, the analyzed sample consisted of 59 participants (Fig 1) with a median age of 59 (50–60) years and a median BMI of 29 (26–36) kg/m^2^, 54% of whom were women.

Ten (16.9%) participants had a history of smoking, with a smoking burden <10 pack-years. The main findings observed in the chest CT examination at the time of acute SARS-CoV-2 infection were as follows: ground-glass opacities (n = 41, 69.5%); consolidation (n = 19, 32.2%); the crazy-paving pattern (n = 14, 23.7%); parenchymal bands (n = 10, 16.9%); the halo sign (n = 7, 11.9%); and subpleural lines (n = 4, 6.8%). Regarding disease extent on CT, 39 (66.1%), 15 (25.4%), and 5 (8.5%) cases were classified as < 25%, 25–50%, and > 50%, respectively. Forty-three patients (72.9%) reported hospitalization at the time of active disease, with a median length of stay of 15 (8–28) days. Eighteen patients (30.5%) were admitted to the intensive care unit, with seven (11.9%) requiring invasive mechanical ventilation. The use of various drugs was reported during the acute phase of COVID-19, including hydroxychloroquine (n = 22, 37.3%), azithromycin (n = 20, 33.9%), ivermectin (n = 19, 32.2%), corticosteroids (n = 18, 30.5%), vitamin C (n = 6, 10.2%), and zinc (n = 6, 10.2%).

**Fig 2:**
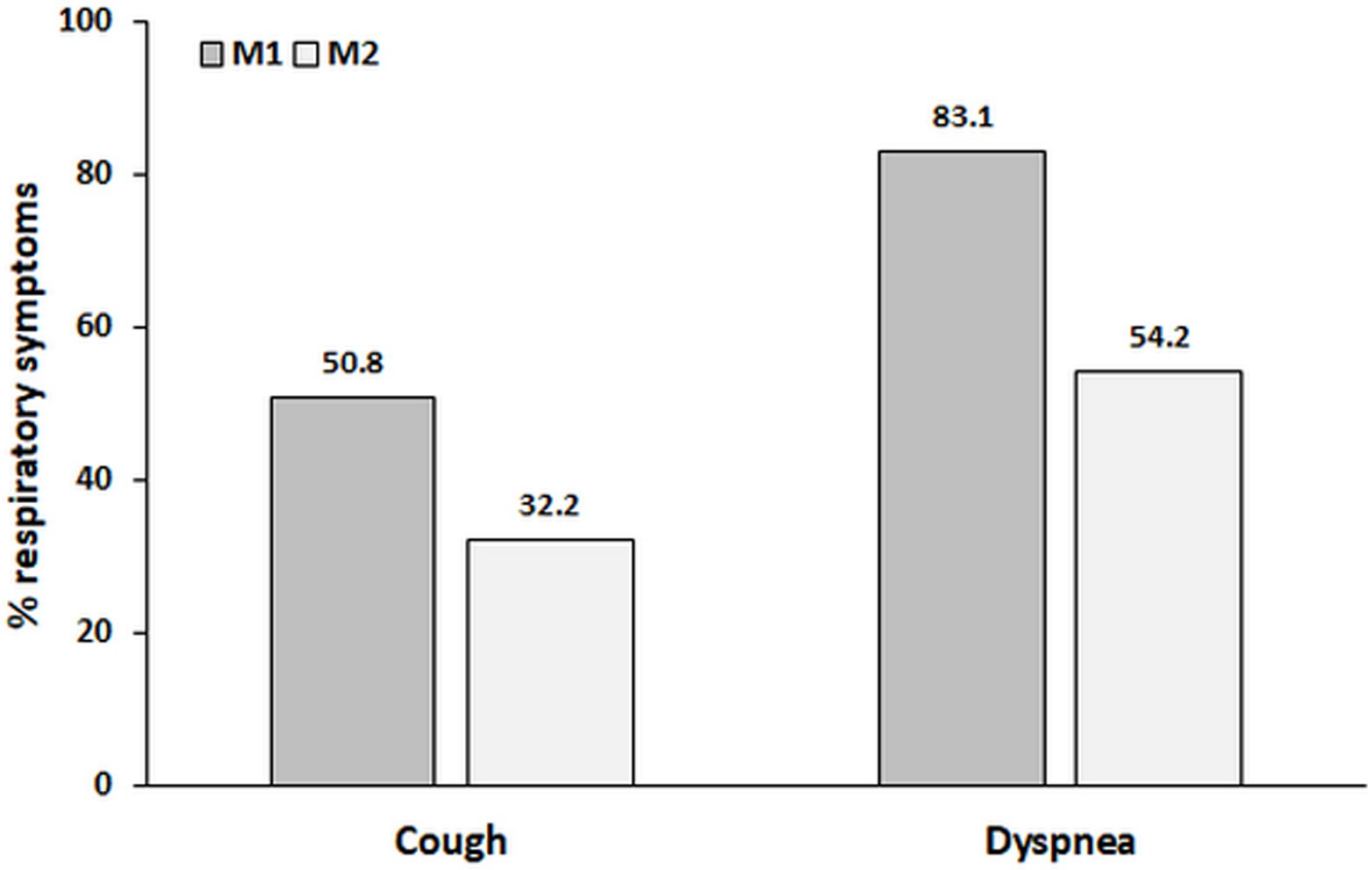
The disease burden detected by respiratory symptoms between the 2nd month (M1) and the 5th month (M2). Significant differences were identified between M1 and M2 for cough (p = 0.012) and dyspnea (p = 0.0004).

The median numbers of days after COVID-19 diagnosis at M1 and M2 were 61 (57–67) and 157 (151–166), respectively. Fifty-nine (100%) participants had cough and/or dyspnea at M1, but this number decreased to 38 (64.4%) at M2 (p = 0.0001). Forty-nine (83.1%) and 32 (54.2%) participants had dyspnea at M1 and M2, respectively (p = 0.0004). Thirty (50.8%) and 19 (32.2%) participants had coughs at M1 and M2, respectively (p = 0.012) (Fig 2). General fatigue was reported by 53 (89.8%) and 38 (64.4%) participants at M1 and M2, respectively (p = 0.0007). The characteristics of the studied sample are shown in Table 1.

**Table 1:** Characteristics of the post-COVID-19 patients.

Participant characteristics	n = 59
**Anthropometric and demographic data**	
Female sex, n (%)	32 (54)
Age, years	59 (50–60)
Weight, kg	80 (70–88)
Height, m	1.64 (1.55–1.72)
BMI, kg/m^2^	29 (26–36)
**Comorbidities**	
Hypertension, n (%)	19 (32.2)
Diabetes, n (%)	12 (20.3)
Chronic heart disease, n (%)	8 (13.5)
Chronic lung disease, n (%)	3 (5.1)

Data are the median (interquartile ranges) or number (%)

BMI = Body mass index.

Regarding PFTs, spirometry was altered in 26 (44.1%) and 20 (33.9%) participants at M1 and M2, respectively, although without statistical significance (p = 0.10). When spirometry was interpreted based on ventilatory patterns, normal examination, restrictive patterns and obstructive patterns were observed in 33 (55.9%), 18 (30.5%) and 8 (13.6%) participants, respectively, at M1 and in 39 (66.1%), 13 (22%) and 7 (11.9%) participants, respectively, at M2 (p = 0.14). On IOS, considering changes in the resistive and reactive parameters, an abnormal result was detected in 52 (88.1%) participants at M1 and in 42 (71.2%) participants at M2 (p = 0.002). A value of R4-R20 >20% was observed in 38 (64.4%) participants at M1 and in 33 (55.9%) participants at M2 (p = 0.30). When comparing M1 with M2, the R4-R20 values were reduced, maintained, and increased in 35 (59.3%), 5 (8.5%), and 19 (32.2%) participants, respectively. Comparisons of the values of the parameters measured by the PFTs between M1 and M2 are shown in Table 2 and Fig 4.

**Table 2:** Comparisons between lung function parameters at 2 months (M1) and 5 months (M2) post-COVID-19.

Lung function parameters	M1	M2	Absolute delta	p value
**Spirometry**				
FVC, % predicted	79 (68–89)	84 (76–92)	4.3 (1.3–8.6)	**<0.0001**
FEV_1_, % predicted	79 (67–88)	85 (77–94)	5.6 (1–8.5)	**<0.0001**
FEV_1_/FVC, %	81 (75–85)	81 (76–85)	-0.44 (-2.4–1.9)	0.36
FEF_25-75%_, % predicted	86 (59–114)	89 (63–127)	6.2 (-2.9–20.3)	**0.003**
**Impulse oscillometry**				
Fres, Hz	20.3 (13.9–27.8)	15.5 (12.2–22)	-3 (-5.9–-1.9)	**<0.0001**
Rm, cm H_2_O/L/s	5.3 (3.7–6.6)	4.1 (3–5.2)	-1.1 (-2–-0.3)	**<0.0001**
R4, cm H_2_O/L/s	6.9 (4.7–9.1)	4.9 (3.7–6.7)	-1.3 (-3–-0.4)	**<0.0001**
R4, % predicted	167 (135–222)	145 (102–165)	-28 (-73–-11)	**<0.0001**
R6, cm H_2_O/L/s	5.9 (3.9–7.8)	4.6 (3.5–5.8)	-1.2 (-2.1–-0.3)	**<0.0001**
R6, % predicted	163 (123–226)	148 (103–171)	-26 (-57–1)	**<0.0001**
R10, cm H_2_O/L/s	5.5 (3.8–6.7)	4.1 (3–5.5)	-1.03 (-1.9–-0.2)	**<0.0001**
R10, % predicted	160 (130–204)	135 (101–169)	-24 (-61–-2)	**<0.0001**
R20, cm H_2_O/L/s	4.4 (3.5–5.9)	3.7 (2.6–4.8)	-0.56 (-1.5–-0.2)	**<0.0001**
R20, % predicted	147 (127–181)	130 (95–160)	-26 (-59–-7)	**<0.0001**
R4-R20, cm H_2_O/L/s	1.8 (0.8–3.5)	1.1 (0.5–2.2)	-0.45 (-1.9–0)	**<0.0001**
AX, cm H_2_O/L	6.3 (3.9–12.5)	3.9 (2.3–7.3)	-2 (-3.4–-0.4)	**<0.0001**

Data are the median (interquartile ranges) or number (%).

FVC = forced vital capacity; FEV_1_ = forced expiratory volume in one second; FEF_25-75%_ = forced expiratory flow during the middle half of the FVC; Fres = resonance frequency; Rm = mean resistance between 4–20 Hz; R4 = resistance at 4 Hz; R6 = resistance at 6 Hz; R10 = resistance at 10 Hz; R20 = resistance at 20 Hz; R4-R20 = heterogeneity of resistance between R4 and R20; AX = area under the reactance curve.

**Fig 3:**
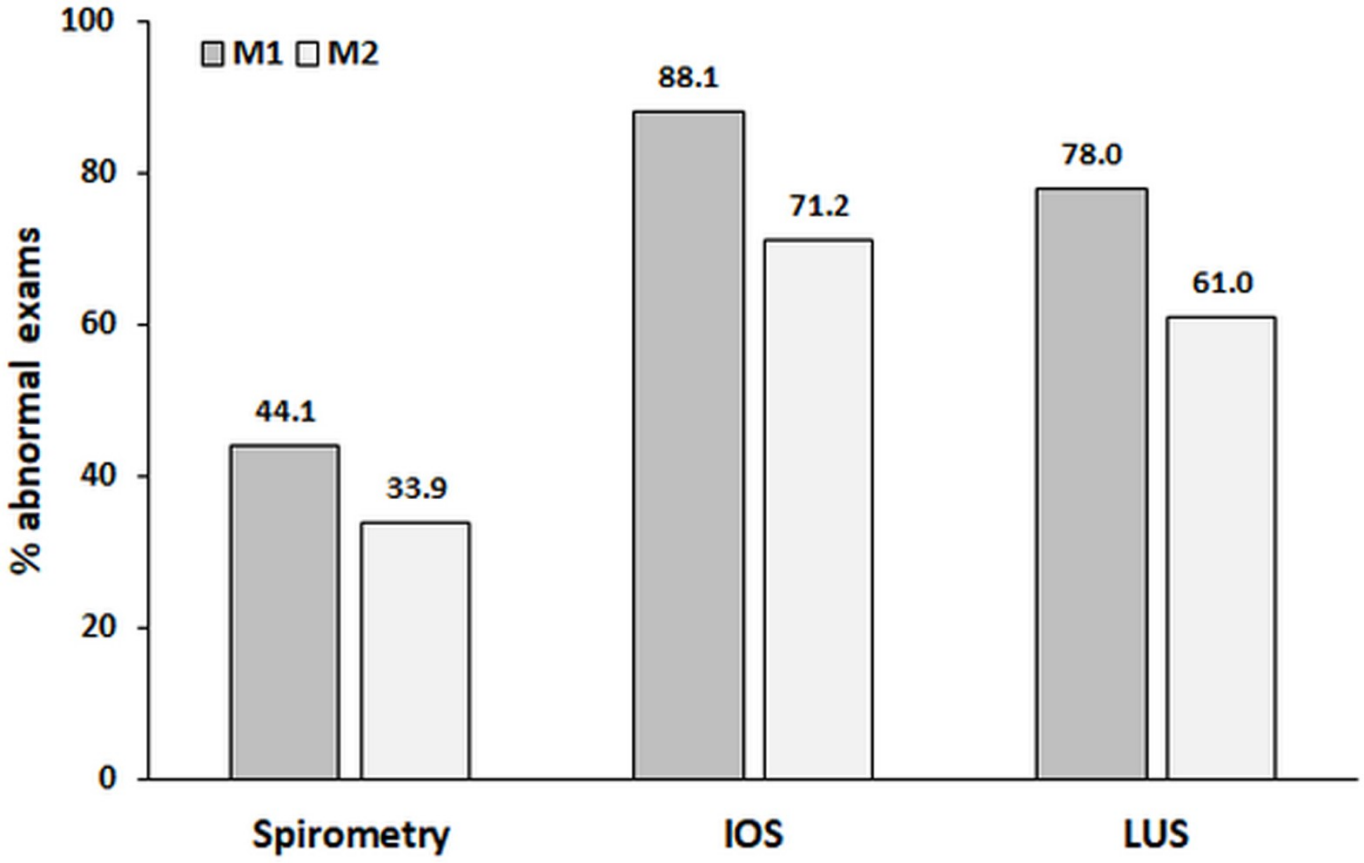
The disease burden detected by abnormalities on spirometry, oscillometry (IOS), and lung ultrasound (LUS) between the 2nd month (M1) and the 5th month (M2). Significant differences were identified between M1 and M2 for IOS and LUS abnormalities (p = 0.002 for both) but not for spirometry abnormalities (p = 0.10).

**Fig 4:**
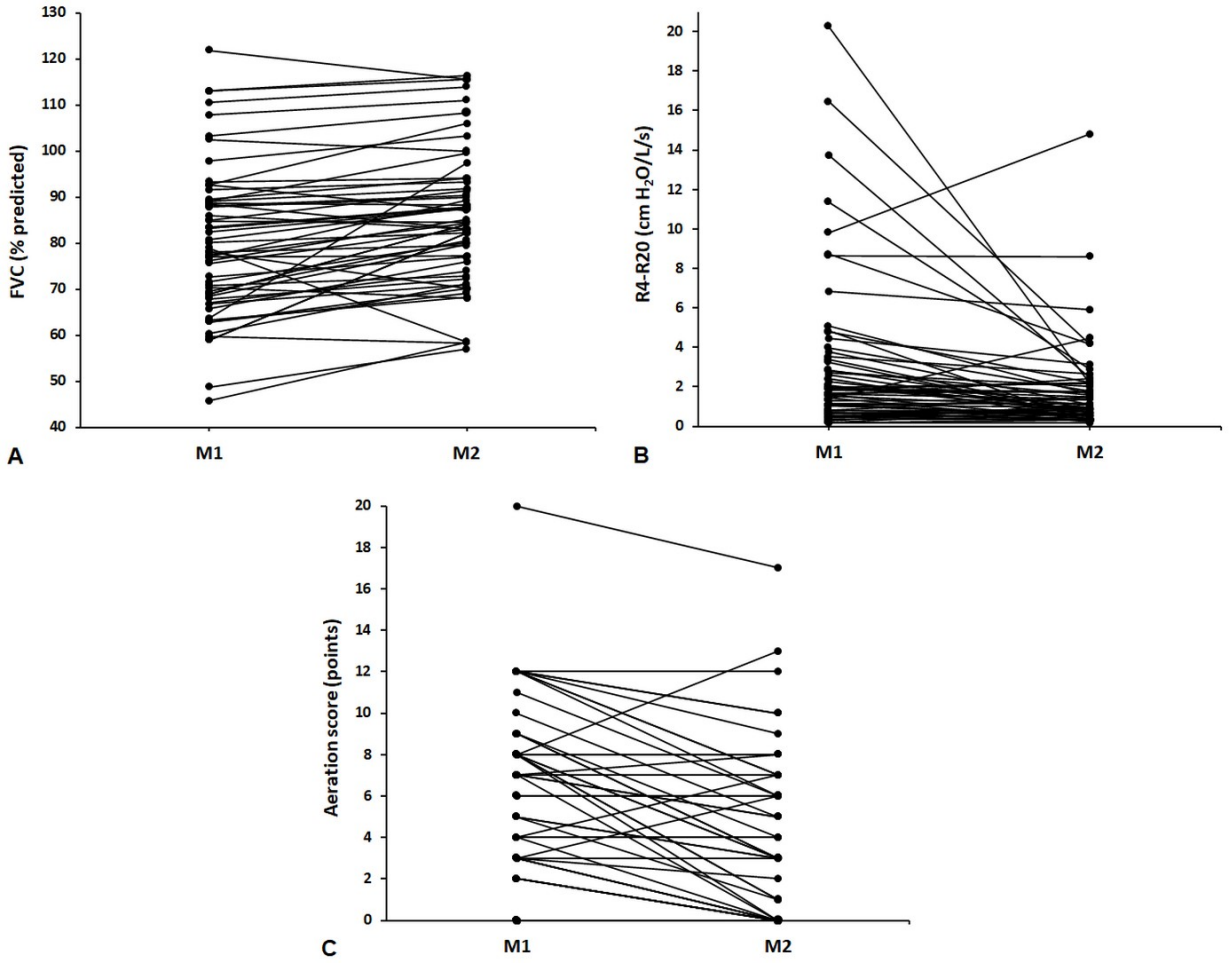
Individual changes in the forced vital capacity (FVC) (A), the heterogeneity of resistance between R4 and R20 (R4-R20) (B), and aeration scores (C) between the 2nd month (M1) and the 5th month (M2).

Finally, we evaluated changes in LUS signals between M1 and M2 (Table 3). Altered LUS was observed in 46 (78%) participants at M1 and in 36 (61%) participants at M2 (p = 0.002). All LUS signals decreased in frequency between M1 and M2 but without statistical significance; however, a statistically significant reduction in the aeration score was found between M1 and M2 [5 (2–8) vs. 3 (0–6) points, p < 0.0001]. The disease burden detected by functional and imaging abnormalities is shown in Figs 3 and 4.

**Table 3:** Comparisons between 2-month (M1) and 5-month (M2) post-COVID-19 lung ultrasound signals.

Lung ultrasound signals	M1	M2	Absolute delta	p value
B-lines >2, n (%)	41 (69.5)	34 (57.8)	-	0.065
Coalescent B-lines, n (%)	13 (22)	12 (20.3)	-	0.99
Subpleural consolidations, n (%)	9 (15.3)	6 (10.2)	-	0.58
Aeration score (points)	5 (2–8)	3 (0–6)	-2 (-4–0)	**<0.0001**

## Discussion

The main result of the present study was that in survivors of COVID-19 with respiratory symptoms, abnormal IOS was the most frequent finding at both the 2nd and 5th months. A decline in the number of abnormal IOS, LUS, and spirometry test results was noted between the 2nd and 5th months; however, this decrease was significant only for IOS and LUS. During this 3-month interval, a significant reduction in respiratory symptoms (cough and/or dyspnea) was identified. On spirometry, almost one-third of the patients presented a restrictive pattern in the 2nd month, the frequency of which decreased to just over 20% in the 5th month. On IOS, almost two-thirds of the patients presented changes compatible with PAD in the 2nd month, and this frequency was reduced to just over half of the patients in the 5th month. On LUS, a statistically significant reduction in the aeration score was found between the 2nd and 5th months post-COVID-19.

COVID-19 symptoms can vary from mild to severe, with considerable mortality rates during the acute phase of the disease [26]. In the present study, we observed a significant reduction in both the frequency of dyspnea and cough in the 3-month interval (between M1 and M2). Despite the high frequency of respiratory symptoms, general fatigue was the most frequent complaint in our sample, a finding that is in agreement with other studies that evaluated post-COVID-19 syndrome [4, 27]. Using social networking tools in a large sample of 2,113 patients 3 months after SARS-CoV-2 infection, Goërtz et al. [4] observed that fatigue and dyspnea were the most prevalent symptoms during acute infection and during follow-up in this population (fatigue: 95% vs. 87%; dyspnea: 90% vs. 71%, respectively). Interestingly, we observed a lower hypertension rate (32.2%) than the average rate reported in Brazilian adults aged 30 years and older (40.7%) [28]. However, the study by Marques et al. [28] used blood pressure measurements to define hypertension unlike our study, which used self-reported diagnoses of hypertension. Because hypertension is one of the main factors associated with death from COVID-19 in Brazilian adults [29], another possible explanation for these differences may be the positive impact of the absence of hypertension on survival after SARS-CoV-2 infection.

In addition to the measurement of DLco, spirometry has been widely used in post-COVID-19 syndrome due to availability; however, its use has raised concerns given the risk of SARS-CoV-2 transmission [10]. In the present study, we observed that spirometry was abnormal in approximately 44% and 34% of patients at M1 and M2, respectively, but the difference was not significant. Interestingly, Orzes et al. [26] showed that most patients with abnormal PFTs (including spirometry and DLco) improved after 3 months but that only 29% had normal values recorded at 6 months, indicating that the recovery of lung function is initially fast but occurs more slowly thereafter. In line with other studies using spirometry in post-COVID-19 syndrome [1, 30–32], we also observed a predominance of the restrictive pattern, which can be explained not only by the interstitial injury induced by SARS-CoV-2 but also by additional mechanisms such as a reduction in respiratory muscle strength and the development of microthrombosis and parenchymal damage [33]. Interestingly, approximately 14% and 12% of the participants in our study had obstructive damage at M1 and M2, respectively, which cannot be explained only by the underlying lung disease present in only 5% of our sample. Certainly, other underlying mechanisms may be involved in the genesis of obstructive damage, including small airway injury, bronchial hyperresponsiveness, and the effect of smoking [34]. In a meta-analysis, Torres-Castro et al. [1] found a 7% prevalence of the obstructive pattern on spirometry.

One of the main findings of our study is the high frequency of patients with changes on IOS at M1—both of resistive and reactive parameters—with a significant reduction in these findings at M2. To the best of our knowledge, this is the first study evaluating the follow-up of patients with post-COVID-19 syndrome at two time points through IOS. Using traditional PFTs at 60 and 100 days after COVID-19 diagnosis, Sonnweber et al. [35] observed impaired lung function in 42% and 36% of individuals at visit 1 and visit 2, respectively, with much lower frequencies of abnormalities than those found on IOS.

Importantly, IOS has been shown to be a sensitive method for the early diagnosis of PAD in various conditions and can detect PAD even before clinical manifestations or spirometric abnormalities appear [14, 36]. In line with these findings, we observed an R4-R20 value >20%—a marker of nonuniformity of airflow distribution and PAD [20, 37]—in almost two-thirds of the cases at M1 and in more than half of the cases at M2, with a pronounced decrease in this parameter in the total sample when the two time points were compared (p < 0.0001). In COVID-19, viral particles were found by electron microscopy in the distal airway mucosal epithelia, potentially resulting in bronchiolitis, a reduction in airway caliber, and even bronchiolar hyperresponsiveness [38]. The mosaic attenuation pattern frequently observed on CT at 4 months after infection by SARS-CoV-2 [30] has been attributed to abnormalities in the distal airways, such as constrictive bronchiolitis with air trapping and secondary reflex vasoconstriction. In a study previously published by our group evaluating 117 post-COVID-19 patients [21], we showed a relationship between PAD diagnosed by IOS and the disease burden assessed by LUS, suggesting that closure of the small airways due to the formation of mucosal plugs and bronchial damage distally in the small airways may result in PAD [34, 39]. Whether COVID-19 sequelae may be driven in part by small airway remodeling remains an open question. SARS-CoV-2 appears to induce characteristic remodeling of the cellular ultrastructure of the respiratory epithelium in a preclinical model [40]. Although almost one-third of our sample showed an increase in the R4-R20 value between M1 and M2—which supposedly may signal PAD exacerbation—we believe that longer-term longitudinal studies are needed to confirm this hypothesis. Nevertheless, we believe that IOS, in addition to being a safe and simple technique to evaluate respiratory sequelae in post-COVID-19 syndrome requiring only tidal breathing maneuvers, can be an instrument to guide therapeutic responses and rehabilitation strategies.

In addition to pulmonary function, diagnostic imaging has emerged as a key component of the evaluation of COVID-19 survivors; thus, LUS can serve as an effective first-line tool for the longitudinal evaluation of post-COVID-19 patients [41]. In fact, we observed abnormal LUS for approximately 80% and 60% of patients at M1 and M2, respectively. The main LUS sign in our study was B-lines >2, detected in approximately 70% of patients at M1 and in approximately 60% of patients at M2. Our findings are similar to those reported in a study by Giovannetti et al. [7], who evaluated the performance of LUS in patients who had hypoxemic acute respiratory failure due to SARS-CoV-2 3 months after discharge and observed signs of residual lung injury in 63.2% of patients through B-lines. Interestingly, B-lines >2 on LUS have been associated with areas of ground-glass opacities on CT in post-COVID-19 patients [9]. The quantification of LUS signs using scoring systems in the follow-up of lung injury is also relevant, especially regarding aeration. We observed a significant reduction in the aeration score between M1 and M2, which points to the sensitivity of LUS signs for the follow-up of this patient population.

Our study has some limitations. First, the sample size was small, and we did not have data on the previous pulmonary function of the patients, precluding determination of the real effect of SARS-CoV-2 infection; however, the comparison between the two study time points (M1 and M2) may facilitate this interpretation. Second, we did not have PFT measurements or LUS signs at the time of acute SARS-CoV-2 infection (baseline). Although CT scans were used to confirm COVID-19 pneumonia, the CT results (baseline) and LUS (M1 and M2) findings were not interchangeable; moreover, PFTs were contraindicated in hospitalized patients with acute SARS-CoV-2 infection [42]. Third, we did not measure pulmonary diffusion, which has been shown to be one of the main functional abnormalities in post-COVID-19 syndrome [1]. Finally, the finding of pathological signs on LUS may be subject to interobserver bias due to the skill of the operator; however, LUS is easy to learn and is less technically demanding than other ultrasound examinations, and all examinations were performed by two operators. Despite these limitations, our study points to the need to follow-up post-COVID-19 patients in both the physiological and imaging domains.

In conclusion, this study shows that in the comparison between the 2nd and 5th months post-COVID-19, an abnormality on IOS was observed in almost 90% of patients with persistent respiratory symptoms, with this frequency decreasing to approximately 70% in the 5th month. Pathological signs on LUS were observed in almost 80% of patients in the 2nd month, with a decrease to approximately 60% of patients in the 5th month. More rarely, changes on spirometry were observed, either in the 2nd or 5th month post-COVID-19. Thus, we believe that IOS and LUS can contribute to the management of patients with post-COVID-19 syndrome, as they are sensitive tests for detecting changes and are safe to perform during the pandemic.
